# Assessing the Consumption of Ultra-Processed Foods in People with Diabetes: Is a Specific NOVA Questionnaire Always Necessary?

**DOI:** 10.3390/nu17010001

**Published:** 2024-12-24

**Authors:** Giovanna D’Abbronzo, Cinzia Quaglia, Giuseppe Di Costanzo, Roberta Testa, Rosalba Giacco, Gabriele Riccardi, Olga Vaccaro, Marilena Vitale

**Affiliations:** 1 Nutrition, Diabetes and Metabolism Research Unit, Department of Clinical Medicine and Surgery, Federico II University, 80131 Naples, Italy; giovanna.dabbronzo@unina.it (G.D.); cinzia.quaglia195@gmail.com (C.Q.); giuseppedicostanzo19@gmail.com (G.D.C.); roberta.testa@unina.it (R.T.); rgiacco@isa.cnr.it (R.G.); riccardi@unina.it (G.R.); ovaccaro@unina.it (O.V.); 2 Institute of Food Sciences, National Research Council, 83100 Avellino, Italy

**Keywords:** UPFs, NOVA classification, food frequency questionnaire, eating habits, type 2 diabetes

## Abstract

Background/Objectives: Despite the accumulating evidence on the detrimental impact of UPFs on health, a common limit of the available studies concerns the instruments used to collect information about the consumption of processed foods. Recently, a specific NOVA-FFQ was proposed for the evaluation of ultra-processed food (UPF) consumption, but it does not allow the simultaneous assessment of energy and nutrient intake. We evaluate the concordance between the NOVA-FFQ and a common questionnaire (EPIC-FFQ) when assessing (1) the intake of foods with different degrees of processing and (2) the relationship between diet composition and cardiometabolic profile. Methods: One hundred and thirty people with diabetes (70 men and 60 women) completed the NOVA-FFQ and the EPIC-FFQ in random order two weeks apart. Anthropometric and major cardiovascular risk factors were measured. Results: Non-significant differences were detected for processed culinary ingredients and processed foods; larger significant differences were observed for minimally processed foods and UPFs, which were somewhat underestimated by the EPIC-FFQ (−24% vs. −21%, respectively; *p* < 0.001). However, Bland–Altman plots showed intraindividual differences between the two questionnaires within an acceptable range, and the intraclass correlation showed a moderate consistency. Furthermore, the energy and nutrient composition of the diet and the metabolic parameters were comparable for people classified in the highest tertile of UPF consumption by either method. Conclusions: The NOVA-FFQ provides more detailed information on the consumption of UPF foods; however, the EPIC-FFQ is a valid alternative, particularly practical when the simultaneous assessment of the overall quality of the diet is needed.

## 1. Introduction

Ultra-processed foods (UPFs) encompass all foods that undergo extensive industrial processes, whether physical, chemical, or biological, such as hydrogenation, hydrolysis, extrusion, and pre-processing by frying. The UPF category also generally includes industrial substances not typically found in home-prepared foods, like maltodextrins, hydrogenated oils, modified starches, flavoring agents, and cosmetic additives such as dyes, emulsifiers, and artificial sweeteners [1].

Consumption of UPFs has been on the rise globally among both adults and children over recent decades [2,3,4,5,6], accounting for up to 31.1% of daily caloric intake in France [7], 56.8% in the UK [8], and 57.9% in the USA [9].

Recent systematic reviews and meta-analyses have revealed associations between UPF consumption and the risk of various chronic conditions, particularly obesity and cardiometabolic outcomes, as well as overall mortality, cancer, frailty, and depressive symptoms [10,11,12,13,14,15]. These studies have been conducted across large cohorts in different countries, such as NutriNet-Santé in France [16,17], SUN in Spain [18,19], and the UK Biobank [20].

Despite the accumulating evidence on the detrimental impact of UPFs on health, a common limit of the available studies concerns the instruments used to collect information about the consumption of processed foods in the target population. A commonly utilized tool is a collection of detailed and repeated 24 h dietary logs. This approach enables the gathering of data on specific brands within a generic food category, facilitating precise UPF classification according to the NOVA system. In the absence of 24 h dietary logs, extensive food frequency questionnaires (FFQs) are also employed to assess the UPF content in a regular diet. However, it is important to underline that neither the 24 h recalls nor the FFQs were designed and validated to estimate the consumption of products undergoing different degrees of food processing; this may lead to misclassifications that could potentially bias the associations between the consumption of UPFs and health outcomes. Taking this into account and prompted by the need to collect information better suited for the categorization of food consumption according to the NOVA classification (e.g., distinguishing between homemade, artisanal, or other types of products, such as biscuits and pizza), Dinu et al. [21] developed and validated in the Italian adult population a NOVA-FFQ specifically designed to estimate the consumption of foods with different degrees of processing according to the NOVA classification. However, this questionnaire does not include information which allows the calculation of the energy intake and nutrient composition of the diet. Therefore, despite the better methodological accuracy regarding the consumption of processed foods, the utilization of this questionnaire in clinical practice may be limited by the need to simultaneously collect information on the composition of the diet with a different FFQ, which makes dietary assessment unpractical and time-consuming, especially in large cohorts.

Against this background, we set up this study to evaluate (1) the concordance between the recently proposed NOVA-FFQ and the European Prospective Investigation into Cancer and Nutrition questionnaire (EPIC-FFQ)—a validated FFQ largely used in epidemiological studies—in relation to the evaluation of the consumption of foods with different degrees of processing, defined according to the NOVA system (i.e., unprocessed and minimally processed foods, MPFs; processed culinary ingredients, PCIs; processed foods, PFs; and ultra-processed foods, UPFs), and (2) the concordance of the two instruments when assessing the association of the intake of foods in the different NOVA classes with the composition of the habitual diet and the cardiovascular risk factor profile. All analyses were performed in a cohort of people with type 2 diabetes.

## 2. Materials and Methods

### 2.1. Participants

From January to July 2023, 130 people with type 2 diabetes (70 men and 60 women) were recruited on a voluntary basis among consecutive patients attending the Diabetes, Nutrition and Metabolism Unit of the Federico II University Hospital, Naples, Italy. The inclusion criteria were an age between 40 and 70 years, a BMI of 25.0–35.0 Kg/m^2^, a glycated hemoglobin level of 7.0–9.0%, and the ability to complete the food frequency questionnaires without assistance. Individuals with compromised kidney function (serum creatinine ≥1.5 mg/dL), those who had a cardiovascular incident in the past six months, those with conditions other than diabetes requiring special dietary treatment, those currently on a weight-loss diet, and those who were pregnant or nursing were excluded from the study. Excluding these specific groups with special nutritional needs or acute conditions which may impact on lifestyle-related habits ensured that the study focused on a relatively homogenous sample, representative of the vast majority of people with type 2 diabetes, thus minimizing the risk of confounding factors that could obscure the relationship between diet and health outcomes. There was no compensation for participating in this study. In order to motivate participation, at the study’s end, a registered dietitian provided each participant with detailed information about their eating habits along with advice on appropriate dietary modifications related to their cardiometabolic profile. The study protocol complied with the Declaration of Helsinki and was approved by the institutional review boards at Federico II University and registered at the ClinicalTrials.gov as NCT03410719. Written informed consent was obtained from all participants.

### 2.2. Study Design

Each participant filled out a concise questionnaire detailing their date of birth, education level, work activities, and regular physical activity. A registered dietitian measured body weight and height using a balance scale (Seca 711, s.r.l.) and a wall-mounted stadiometer (Seca 720, s.r.l.), respectively. The body mass index (BMI) was calculated from the recorded weight and height (kg/m^2^). Waist circumference and sitting blood pressure were assessed following a standard procedure. Blood samples were collected in the morning after an overnight fast, and all biochemical analyses were conducted in the hospital’s central laboratory. Total cholesterol, HDL cholesterol, and triglycerides were measured using standard techniques. LDL cholesterol was calculated using the Friedewald equation, but only for triglyceride values below 400 mg/dL [22]. Glycated hemoglobin (HbA1c) was measured using high-performance liquid chromatography, standardized according to IFCC guidelines. All participants completed the EPIC-FFQ and the NOVA-FFQ in random order, two weeks apart. They were strongly encouraged to maintain their usual dietary habits during this period. Any significant dietary changes due to holidays, illness, travel, or other reasons were identified by the dietitian through a brief survey with each participant.

### 2.3. EPIC Food Frequency Questionnaire (EPIC-FFQ)

The European Prospective Investigation into Cancer and Nutrition (EPIC) questionnaire, tailored for the Italian population, is a validated tool commonly used in large-scale epidemiological research [23,24]. The questionnaire comprises 248 items covering 188 different foods, including the quantity and type of fat used as a condiment or added after cooking. Foods are categorized into 83 pre-defined groups based on similar nutrient characteristics or culinary uses. Participants report the exact frequency of consumption (per day, week, month, or year) for each item and the portion size by selecting images depicting small, medium, and large servings, with additional options for portion variations (e.g., “smaller than the small portion” or “between the small and medium portion”). Questionnaires that were incomplete or contained implausible data (e.g., an energy intake less than 800 or greater than 5000 Kcal/day) were excluded from the analysis. A specific software package, developed by the Epidemiology and Prevention Unit at Fondazione IRCCS, Istituto Nazionale dei Tumori, Milan, was used to convert the dietary data from the questionnaire into average daily food amounts (g/day). The analysis of each FFQ was conducted using a computer program that employs the Italian Food Composition Tables (FCTs) for energy and nutrient assessment.

### 2.4. NOVA Food Frequency Questionnaire (NOVA-FFQ)

The NOVA-FFQ (Food Frequency Questionnaire based on the NOVA classification) is a dietary assessment tool that categorizes food items based on their level of food processing, following the NOVA classification system. The reproducibility and validity of this questionnaire to assess food consumption based on the NOVA classification in adults were tested by Dinu et al. by administrating the NOVA-FFQ to 110 subjects on two different occasions and comparing it with a weighted dietary record (WDR) [21]. The NOVA-FFQ provided 94 items on frequency of consumption and serving sizes divided into nine categories: (1) fruits and nuts; (2) vegetables and legumes; (3) cereals and tubers; (4) meat and fish; (5) milk, dairy products, and eggs; (6) oils, fats, and seasonings; (7) sweets and sweeteners; (8) beverages; and (9) other. The habitual portion size was evaluated by selecting one out of six options. As reference serving sizes, the NOVA-FFQ used those indicated in the “Quantitative standards for portions” of the Italian Society of Human Nutrition (Società Italiana di Nutrizione Umana (SINU) 2014). Metadieta software (Meteda s.r.l., version 4.5; Italy) was used by a trained dietitian to convert dietary data from the questionnaire into average daily amounts of foods (g/day). For each participant, the average energy (kcal) and nutrient intake (g/day) of protein, fat, saturated fatty acids, cholesterol, carbohydrates, dietary fiber, and alcohol were computed by the software according to the Italian Food Composition Databases. Data were exported to Excel for statistical analyses.

### 2.5. Measurement of MPFs, PCIs, PFs, and UPFs

The completed FFQs (both EPIC and NOVA) were reviewed by an expert dietitian and corrected for missing items or misrecorded serving sizes by interviewing the study participant. To estimate the consumption of MPFs, PCIs, PFs, and UPFs, we used the NOVA classification, which includes four categories representing different levels of processing: (i) fresh or minimally processed foods (e.g., fruit, meat, and milk); (ii) processed culinary ingredients (e.g., oils and butter); (iii) processed foods (e.g., canned fish); and (iv) UPFs containing predominantly industrial substances and little or no whole foods (e.g., carbonated drinks, processed meat, and snacks). The amount of each food consumed (g/day) by each study participant was summed up in the appropriate group of the NOVA classification.

### 2.6. Sample Size and Statistical Analysis

The aim of this study was to determine whether the two methods were correlated with an intraclass correlation coefficient (ICC) of ≥0.70 for UPF consumption. The required sample size to detect a 10% difference in the consistency between the two methods was calculated to be 120 participants (accounting for a 10% drop-out rate), with a type I error α = 0.05 and a type II error β = 0.1 (80% power). Data are presented as means ± standard deviations or percentages as appropriate. Paired Student’s *t*-tests were used to compare the means of MPFs, PCIs, PFs, and UPFs obtained using the two methods. A repeated-measures Bland–Altman analysis was employed to assess the relative bias (mean difference) and random error (1.96 standard deviations (SDs) of the intra-individual difference) between the EPIC-FFQ and NOVA-FFQ methods. The intraclass correlation coefficient (ICC) was utilized to evaluate the consistency between the two methods. According to Koo and Li [25], consistency between the methods can be poor (ICC below 0.50), moderate (ICC between 0.50 and 0.75), good (ICC between 0.75 and 0.90), or excellent (ICC above 0.90). Finally, to investigate the relationship between UPF consumption (evaluated using either EPIC-FFQ or NOVA-FFQ), the energy and nutrient composition of the diet, and the cardiometabolic profile of participants, we compared these parameters in individuals classified in the highest tertile of UPF intake according to each method. All statistical analyses were conducted using SPSS version 28 (IBM Inc., Armonk, NY, USA), with *p*-values <0.05 considered statistically significant.

## 3. Results

One hundred and thirty people with type 2 diabetes participated in the study (70 men and 60 women), with a mean age of 67.5 ± 10.4 years, a mean BMI of 29.9 ± 6.1 kg/m^2^, and a mean waist circumference of 104.7 ± 16.7 cm. All of them had good glycemic control, as indicated by plasma glucose (94.3 ± 54.9 mg/dL) and HbA1c (6.8 ± 2.3%) values. The level of education was a university degree for 50 participants and a secondary school diploma for 80 participants.

The daily intakes of processed food in the study population estimated according to the EPIC-FFQ or the NOVA-FFQ are reported in Table 1.

**Figure 1:**
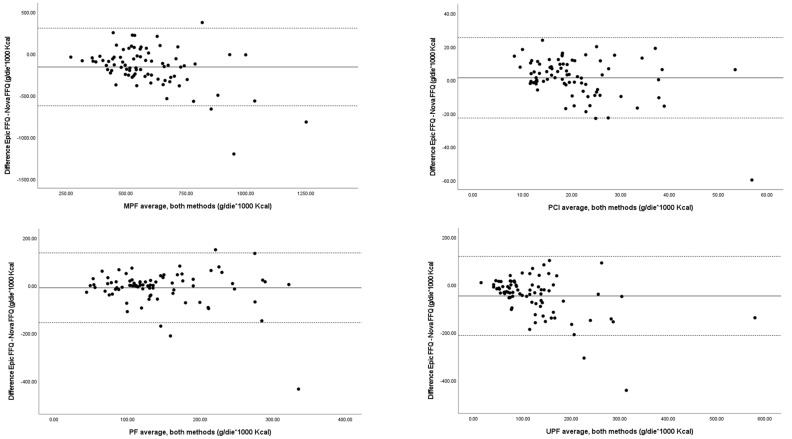
Repeated-measures Bland–Altman plots of the differences in intakes measured according to the EPIC-FFQ and NOVA-FFQ for unprocessed and minimally processed foods (MPFs), processed culinary ingredients (PCIs), processed foods (PFs), and ultra-processed foods (UPFs). The solid lines indicate the mean differences, while the dashed lines indicate the 95% limits of agreement (LOAs) (1.96 SDs).

Only minor and non-statistically significant differences between the EPIC-FFQ and the NOVA-FFQ were observed for PCIs (21.5 ± 8.6 vs. 20.1 ± 12.9 g/1000 kcal/day, +1.4%, *p* = 0.309) and PFs (138.9 ± 70.0 vs. 147.9 ± 82.6 g/1000 Kcal/day, −9.0%, *p* = 0.280), whereas larger differences were observed for the consumption of MPFs and UPFs, which were somewhat underestimated by the EPIC-FFQ (MPFs: 512.2 ± 155.1 vs. 670.3 ± 248.1 g/1000 kcal/day, −24%, *p* < 0.001; UPFs: 101.8 ± 74.0 vs. 148.0 ± 109.2 g/1000 Kcal/day, −21%, *p* < 0.001). Although statistically significant, these differences were of modest magnitude, as suggested by the Bland–Altman analyses, which indicated that intraindividual differences between the two questionnaires were within acceptable ranges for MPF, PCI, PF, and UPF intakes, with very few outliers (Figure 1).

In agreement with these analyses, the intraclass correlation coefficient (ICC), used to assess the consistency between the two methods, indicated a moderate consistency, similar for all food groups (Table 2).

The ICCs were, in fact, 0.510 for MPFs (*p* < 0.001), 0.541 for PCIs (*p* < 0.001), 0.689 for PFs (*p* < 0.001), and 0.741 for UPFs (*p* < 0.001).

To further evaluate the clinical impact of the use of the EPIC-FFQ or NOVA-FFQ, we compared the energy and nutrient composition of the diet, as well as the cardiovascular risk factor profile, for people classified in the upper sex-specific tertile of consumption of UPFs according to either questionnaire. Interestingly, 84% of those classified in this group according to the NOVA-FFQ were classified in the same group according to the EPIC-FFQ, and the energy and nutrient compositions of the habitual diets, as well as the metabolic parameters, were very similar in the two groups (Table 3 and Table 4).

## 4. Discussion

In the present study, for the first time, we comparatively evaluated the performance of the EPIC-FFQ—a questionnaire developed to evaluate habitual diets—and the NOVA-FFQ—specifically designed to assess the intake of differently processed foods as classified by the NOVA system (i.e., MPCs, PCIs, PFs, and UPFs)—as methods for the estimation of the intake of UPFs. The results show a good agreement between the two methods in the assessment of PCI and PF intake, whereas for MPFs and UPFs the EPIC-FFQ tended to underestimate consumption. This was due to the fact that, not unexpectedly, the number of foods included in the MPF and UPF groups were lower in the EPIC-FFQ compared to the NOVA-FFQ. In particular, the NOVA-FFQ is much more detailed regarding all food that undergoes intense industrial physical, chemical, or biological processes and ingredients that are not usually found in home-made foods. However, although statistically significant, the observed differences were of very modest magnitude and unlikely to have clinical impact. The Bland–Altman graphs indicated, in fact, that intraindividual differences between the two methods were within acceptable ranges, with very few outliers. These outliers very likely arose from misreporting or inconsistent reporting methods for individual dietary habits. To reduce the impact of outliers and intraindividual variations, it may be necessary to ensure that participants accurately report their intake using repeated measures.

Furthermore, the intraclass correlation coefficients show a similar grade of consistency between the two methods for all groups of foods, as classified by the NOVA system, thus suggesting that the EPIC-FFQ is a valid tool for the assessment of UPF consumption at the population level.

It is also relevant to underline that an important objective of the NOVA classification was the identification of food groups that had a different impact on the habitual diet composition and on the cardiovascular risk factor profile. Therefore, in the comparison between the two questionnaires, it was important to assess whether a small difference in the estimated consumption of UPFs had any impact on the assessment of the relationship between UPF consumption, the composition of the diet, and the cardiometabolic profile of the participants. The results of our study indicate that the energy and nutrient composition of the habitual diet, as well as the metabolic parameters of people with elevated UPF consumption, such as those classified in the upper tertile of UPF consumption, were not significantly different whether one or the other FFQ was used, largely due to the fact that 84% of those people classified in the upper tertile of UPF consumption with the NOVA-FFQ were also classified in the upper tertile of UPF consumption with the use of the EPIC-FFQ.

Overall, the study results suggest that the two methods (i.e., EPIC-FFQ and NOVA-FFQ) provide reasonably comparable estimates of the amount of food consumed in the four groups of the NOVA classification and, therefore, that both of them can be used in nutritional research.

They are, however, not superimposable; by definition, the NOVA-FFQ gives a more accurate estimate of the amount of UPFs consumed but, unlike the EPIC-FFQ, does not allow the simultaneous assessment of the energy and nutrient composition of the habitual diet, and, therefore, the EPIC-FFQ is a suitable alternative to NOVA-FFQ, particularly practical when the simultaneous assessment of the overall quality of the diet is needed.

To date, all studies published on the NOVA classification system highlight a significant limitation: UPFs are estimated using questionnaires that are not specifically designed for this purpose. These studies suggest that future applications of the NOVA classification system to dietary data would benefit from collecting more detailed information, such as brand names, ingredient lists from packaged foods, and whether meals were prepared at home from minimally processed ingredients or purchased as processed, prepared, or ready-to-eat meals. In this context, the study’s findings are important, as they emphasize that when the EPIC-FFQ (or another validated food frequency questionnaire) is used, any potential misclassification related to the NOVA classification system has minimal, if any, impact on the interpretation of the results. This is particularly important given the large use of the EPIC-FFQ or other non-specific NOVA questionnaires in completed and ongoing epidemiological studies.

Researchers considering using this approach for future studies should be aware of its potential limitations. Firstly, the food frequency questionnaires rely on self-reported dietary data, which may introduce some challenges in interpreting the results [26]. Nonetheless, the EPIC-FFQ used in our study is among the least-biased self-report tools available, and the standardized methodology has proven effective in generating acceptable intake estimates and in assessing the contribution of different food groups to the overall diet. There is a possibility of underreporting foods high in caloric sweeteners or fats, such as desserts and sweet baked goods, which could lead to an underestimation of the dietary contribution of ultra-processed foods. It is also important to note that the study population consisted of individuals with type 2 diabetes, which means the results may not be applicable to other populations. Additionally, the generalizability of the NOVA questionnaire results is limited, as the participants were a random sample of 18–65-year-olds living in Italy. For studies involving different age groups or conducted in other countries, the questionnaire should be adjusted in terms of food items and portions and then revalidated. Finally, the study was conducted in one center of the Campania region in Italy; therefore, the results do not necessarily fully apply to residents of other regions with different cultures and different dietary habits. In fact, while monocentric studies provide valuable insights, some limitations in terms of generalizability also apply due to factors such as the limited demographic diversity of the study participants and cultural differences between regions, underscoring the need for multicenter research. In this respect, multicenter studies enhance the robustness and applicability of findings, ensuring that the conclusions drawn are more broadly relevant and can inform policies or interventions in diverse settings. Future studies should therefore consider adopting a multicenter design to better capture the complexity and diversity of populations.

## 5. Conclusions

In conclusion, our study demonstrates that both the NOVA-FFQ and the EPIC-FFQ are appropriate to assess the intake of UPFs in clinical and research settings, as the two methods provide reasonably comparable estimates of the amount of food consumed in the four groups of the NOVA classification. Although the specific NOVA questionnaire provides more accurate information on the consumption of UPF foods, the EPIC-FFQ is a valid alternative, particularly useful when—as it is often the case—information is needed not only on the degrees of processing but also on the energy and nutrient composition of the habitual diet. This would permit the practical difficulty of utilizing two different questionnaires (i.e., NOVA in combination with a food frequency questionnaire) to collect information relevant for both the NOVA classification and the assessment of the overall quality of the diet to be overcome.

## Figures and Tables

**Table 1:** Daily intake of foods in the four NOVA classes according to the EPIC-FFQ or NOVA-FFQ.

	EPIC-FFQ	NOVA-FFQ	Difference (%) EPIC-FFQ vs. NOVA-FFQ	*p*-Value
MPFs (g/1000 Kcal)	512.2 ± 155.1	670.3 ± 248.1	−24	<0.001
PCIs (g/1000 Kcal)	21.5 ± 8.6	20.1 ± 12.9	+1	0.309
PFs (g/1000 Kcal)	138.9 ± 70.0	147.9 ± 82.6	+6	0.280
UPFs (g/1000 Kcal)	101.8 ± 74.0	148.0 ± 109.2	−31	<0.001

M ± SD. MPFs: unprocessed and minimally processed foods; PCIs: processed culinary ingredients; PFs: processed foods; UPFs: ultra-processed foods.

**Table 2:** Intraclass correlation coefficient for the estimated daily intake of foods in the four NOVA classes according to the EPIC-FFQ and NOVA-FFQ.

	ICC	*p*-Value	Grade of Consistency
MPFs (g/1000 Kcal)	0.510	<0.001	Moderate
PCIs (g/1000 Kcal)	0.541	<0.001	Moderate
PFs (g/1000 Kcal)	0.689	<0.001	Moderate
UPFs (g/1000 Kcal)	0.741	<0.001	Moderate

ICC: intraclass correlation coefficient; MPFs: unprocessed and minimally processed foods; PCIs: processed culinary ingredients; PFs: processed foods; UPFs: ultra-processed foods.

**Table 3:** Energy and nutrient compositions of the diets of the study participants belonging to the upper tertile of UPF consumption evaluated according to EPIC-FFQ or NOVA-FFQ.

	EPIC-FFQ	NOVA-FFQ	*p*-Value
Energy (Kcal/day)	1635.1 ± 303.3	1754.9 ± 437.5	0.248
Proteins (g)	72.7 ± 14.7	75.4 ± 16.3	0.518
Proteins (% of TE)	17.9 ± 2.8	17.4 ± 2.2	0.459
From animal food sources (g)	50.82 ± 13.5	52.8 ± 12.9	0.576
From animal food sources (% of TE)	12.6 ± 3.3	12.3 ± 2.6	0.657
From vegetable food sources (g)	21.7 ± 6.2	22.4 ± 6.8	0.669
From vegetable food sources (% of TE)	5.3 ± 1.1	5.1 ± 1.2	0.626
Total fat (g)	68.4 ± 18.1	72.5 ± 21.5	0.450
Total fat (% of TE)	37.4 ± 5.2	37.2 ± 6.4	0.929
SAFAs (g)	21.1 ± 6.0	22.8 ± 7.0	0.343
SAFAs (% of TE)	11.6 ± 2.3	11.7 ± 2.2	0.847
MUFAs (g)	30.9 ± 8.6	32.6 ± 10.5	0.536
MUFAs (% of TE)	16.8 ± 2.6	16.7 ± 3.7	0.856
PUFAs (g)	10.3 ± 3.2	10.9 ± 3.8	0.497
PUFAs (% of TE)	5.6 ± 1.1	5.6 ± 1.4	0.924
Cholesterol (mg/1000 Kcal)	284.3 ± 75.6	295.7 ± 65.6	0.557
Carbohydrates (g)	189.9 ± 40.0	205.0 ± 53.1	0.243
Carbohydrates (% of TE)	43.7 ± 6.1	43.9 ± 6.1	0.912
Soluble carbohydrates (g)	68.1 ± 21.5	78.2 ± 33.5	0.193
Soluble carbohydrates (% of TE)	15.9 ± 5.2	16.8 ± 6.2	0.558
Fiber (g)	16.5 ± 3.8	17.7 ± 5.6	0.332
Fiber (g/1000 Kcal)	10.1 ± 1.9	10.1 ± 1.9	0.959
Alcohol (g)	2.27 ± 3.4	4.3 ± 11.2	0.366

M ± SD.

**Table 4:** General characteristics and metabolic profile of the study participants belonging to the upper tertile of UPF consumption evaluated according to the EPIC-FFQ or the NOVA-FFQ.

	EPIC-FFQ	NOVA-FFQ	*p*-Value
Age (years)	62.5 ± 10.3	64.5 ± 10.1	0.474
BMI (Kg/m^2^)	31.2 ± 7.2	30.7 ± 6.4	0.797
Waist circumference (cm)	107.8 ± 19.9	107.1 ± 19.0	0.899
Systolic blood pressure (mmHg)	79.5 ± 9.0	81.3 ± 10.3	0.507
Diastolic blood pressure (mmHg)	85.0 ± 67.9	85.3 ± 69.0	0.988
HbA1c (%)	7.0 ± 1.0	7.1 ± 1.2	0.893
Total cholesterol (mg/dL)	158.7 ± 38.3	154.8 ± 37.2	0.711
LDL-c (mg/dL)	84.0 ± 34.0	80.8 ± 32.9	0.731
Triglycerides (mg/dL)	122.5 ± 49.0	106.8 ± 43.1	0.216
HDL-c (mg/dL)	48.7 ± 17.1	50.7 ± 17.1	0.668
Creatinine (mg/dL)	1.0 ± 0.3	1.2 ± 0.7	0.177

M ± SD.

## Data Availability

The datasets generated during and/or analyzed during the current study are available from the corresponding author on reasonable request due to ethical reasons.
